# Insights into Activation Mechanisms of Store-Operated TRPC1 Channels in Vascular Smooth Muscle

**DOI:** 10.3390/cells9010179

**Published:** 2020-01-10

**Authors:** Miguel A. S. Martín-Aragón Baudel, Jian Shi, William A. Large, Anthony P. Albert

**Affiliations:** 1 Department of Pharmacology, University of California, Davis, CA 95615, USA; martinaragon@ucdavis.edu; 2 LIGHT Laboratories, Leeds Institute of Cardiovascular and Metabolic Medicine, University of Leeds, Leeds LS2 9JT, UK; J.Shi1@leeds.ac.uk; 3 Vascular Biology Research Centre, Molecular and Clinical Research Institute, St. George’s, University of London, London SW17 0RE, UK; largew@sgul.ac.uk

**Keywords:** TRPC1, PKC, PIP_2_, Gq, PLC, MARCKS, STIM1, Orai1, store-operated channels, vascular smooth muscle

## Abstract

In vascular smooth muscle cells (VMSCs), the stimulation of store-operated channels (SOCs) mediate Ca^2+^ influx pathways which regulate important cellular functions including contraction, proliferation, migration, and growth that are associated with the development of vascular diseases. It is therefore important that we understand the biophysical, molecular composition, activation pathways, and physiological significance of SOCs in VSMCs as these maybe future therapeutic targets for conditions such as hypertension and atherosclerosis. Archetypal SOCs called calcium release-activated channels (CRACs) are composed of Orai1 proteins and are stimulated by the endo/sarcoplasmic reticulum Ca^2+^ sensor stromal interaction molecule 1 (STIM1) following store depletion. In contrast, this review focuses on proposals that canonical transient receptor potential (TRPC) channels composed of a heteromeric TRPC1/C5 molecular template, with TRPC1 conferring activation by store depletion, mediate SOCs in native contractile VSMCs. In particular, it summarizes our recent findings which describe a novel activation pathway of these TRPC1-based SOCs, in which protein kinase C (PKC)-dependent TRPC1 phosphorylation and phosphatidylinositol 4,5-bisphosphate (PIP_2_) are obligatory for channel opening. This PKC- and PIP_2_-mediated gating mechanism is regulated by the PIP_2_-binding protein myristoylated alanine-rich C kinase (MARCKS) and is coupled to store depletion by TRPC1-STIM1 interactions which induce Gq/PLCβ1 activity. Interestingly, the biophysical properties and activation mechanisms of TRPC1-based SOCs in native contractile VSMCs are unlikely to involve Orai1.

## 1. Introduction

In vascular smooth muscle cells (VSMCs), neurotransmitters and hormones such as noradrenaline, adrenaline, angiotensin II (Ang II), and endothelin 1 (ET-1) produce an increase in cytosolic Ca^2+^ concentration (Ca^2+^_i_) (see [1,2,3,4,5,6,7] for reviews on Ca^2+^ signaling mechanisms in smooth muscle). This rise in Ca^2+^_i_ is important for initiating vasoconstriction, which determines vascular resistance and blood pressure and ultimately regulates blood flow to our tissues and organs. In addition, an excessive increase in Ca^2+^_i_ is linked to cell proliferation, migration and growth, phenotypes which are involved in the development of vascular disease. Therefore, understanding cellular pathways which mediate increases in Ca^2+^_i_ may help to identify future therapeutic targets for conditions such as hypertension and atherosclerosis.

Vasoconstrictors induce a rise in Ca^2+^_i_ by activating the classical phosphoinositol signaling pathway involving: stimulation of Gq-protein-coupled receptors, phospholipase C (PLC) activity, phosphatidylinositol 4,5-bisphosphate (PIP_2_) hydrolysis, and inositol 1,4,5-trisphosphate (IP_3_) and diacylglycerol (DAG) generation [1,2,3,4,5,6,7]. IP_3_ mediates an increase in Ca^2+^_i_ by causing the release of Ca^2+^ from sarcoplasmic reticulum (SR) Ca^2+^ stores, and this subsequent rise in Ca^2+^_i_ and DAG-mediated pathways induce Ca^2+^ influx from the extracellular medium. A component of this Ca^2+^ influx occurs through activation of voltage-gated Ca^2+^ channels (VGCCs), but there is also a significant contribution from voltage-independent Ca^2+^-permeable non-selective cation channels. Stimulation of Ca^2+^-permeable cation channels are thought to mediate Ca^2+^ entry pathways through direct Ca^2+^ influx, and through Na^+^ influx which leads to depolarization and activation of voltage-gated Ca^2+^ channels and stimulation of the Na^+^/Ca^2+^ exchanger in reverse mode.

There is substantial evidence that Ca^2+^-permeable non-selective cation channels classified as receptor-operated (ROCs) and store-operated (SOCs) are expressed in VSMCs [1,2]. ROCs are defined as ion channels activated by receptor stimulation independently of IP_3_-mediated depletion of SR Ca^2+^ stores, whereas SOCs are activated by pathways coupled to depletion of SR Ca^2+^ stores but not by the subsequent increase in Ca^2+^_i_. These definitions infer that stimulation of Gq-protein-coupled receptors will activate both ROCs and SOCs. In addition, it is proposed that SOCs may be activated by Gq-protein-coupled receptor stimulation independently of store depletion, hence SOCs may also function as ROCs [8]. Together, these findings make it difficult to study ROCs and SOCs independently of each other using macroscopic measurements such as whole-cell patch clamp and Ca^2+^ signal recordings but possible using single channel recordings [9,10,11]. Moreover, SOCs can be selectively activated by depleting SR Ca^2+^ stores in the absence of receptor stimulation using SR Ca^2+^-ATPase inhibitors (e.g., cyclopiazonic acid (CPA) and thapsigargin), and high (e.g., 1,2-bis(o-aminophyoxy)ethane-*N*,*N*,*N*’,*N*’-tetraacetic acid (BAPTA)) and low affinity (e.g., *N*,*N*,*N*’,*N*’,-tetrakis(2-pyridmethyl)-1,2-ethnediamine (TPEN)) Ca^2+^ chelators to passively deplete Ca^2+^_i_ and SR Ca^2+^ levels respectively.

This article reviews our current understanding of the biophysical properties, molecular composition, and activation mechanisms of SOCs in VSMCs, in particular, it focuses on recent studies from our laboratory which have described a novel activation pathway of SOCs composed of canonical transient receptor potential 1 (TRPC1) proteins in native contractile VSMCs. In the majority of these studies SOCs were activated by SR Ca^2+^ ATPase inhibitors or Ca^2+^ chelators to prevent complications from activation of ROCs, although in the final section we discuss the potential physiological significance of this store-operated activation pathway in vasoconstrictor-induced TRPC1-based SOCs.

## 2. Biophysical Properties and Molecular Composition of SOCs in VSMCs

It is well-established that archetypal SOCs, termed calcium release-activated channels (CRACs), are characterized by high Ca^2+^ permeabilities (PCa^2+^:PNa^+^ > 1000:1), pronounced inward rectification with reversal potentials (E_rev_) greater than +50 mV, unitary conductances in the fS range, and are composed of Orai1 proteins (see [12] for review on properties and functions of Orai channels and their associated activation mechanisms). The fundamental activation mechanism of Orai1-based CRACs is also clearly outlined, with an essential role for stromal interaction molecule 1 (STIM1) which senses Ca^2+^ levels within ER/SR Ca^2+^ stores and following store depletion undergoes oligmerisation and translocation to cytosolic surface of the plasma membrane where it interacts with Orai1 to induce channel assembly and gating.

However, it is apparent that many cell types express SOCs with very different characteristics to Orai1-based CRACs such as much lower Ca^2+^ permeabilities, relatively linear or outward rectification, and considerably larger unitary conductances (see [13] for review on role of TRPC proteins in mediated SOCs). Given the properties of these SOCs, they are unlikely to be composed of Orai proteins. The molecular composition of these SOCs is controversial but there is increasing evidence that they are formed by the TRPC family of Ca^2+^-permeable non-selective cation channels (TRPC1–C7) [13]. These TRPC-based SOCs are likely to form a diverse group of channels with differing expression, properties, and functions since TRPC subunits form heterotetrameric structures [7,13]. There is further controversy over whether TRPC proteins form SOCs due to limited understanding, unlike with Orai proteins, of how store depletion couples to channel activation that is an essential requirement when defining SOCs. These issues are beginning to be unraveled by findings which suggest that store depletion activates TRPC-based SOCs through diverse STIM1-mediated processes including direct interactions between TRPC and Orai1 proteins, activation of Orai1-based CRACs as a prerequisite for TRPC1 opening, and direct interactions between TRPC and STIM1 [13,14]. For example, in overexpression studies it is proposed that direct interactions between STIM1 and TRPC channels govern activation [13,14,15,16,17,18], whereas elegant studies in salivary glands have shown that activation of TRPC1-based SOCs require prior activation of Orai1-based CRACs to induce insertion of TRPC1 subunits into the plasma membrane, with both activation of Orai1-based CRACs and TRPC1-based SOCs coupled to store depletion by STIM1 (see [19] for review on activation mechanisms of TRPC1-based SOCs in salivary glands). The studies highlighted in this review indicate that activation of STIM1-mediated TRPC1-based SOCs in native contractile VSMCs involves a Gq/PLCβ1 pathway and interactions between protein kinase C (PKC) activity and PIP_2_ which do not require Orai1.

The molecular composition and activation mechanisms of SOCs in VSMCs reflects the controversies highlighted above as there is significant evidence to indicate that both Orai1-based CRACs and TRPC-based SOCs are present in VSMCs, but that these channels are differentially expressed according to cell phenotype. In freshly isolated VSMCs or primary cultured VSMCs maintained in low serum, conditions in which VSMCs retain their native contractile phenotype, evidence suggests that SOCs have biophysical properties which resemble TRPC-based SOCs and not Orai1-based CRACs with relatively linear, S-shaped, or slightly outward rectification, low Ca^2+^ permeabilities (PCa^2+^:PNa^+^ of 1–50), and unitary conductances of 2–3 pS and 2–8 pS in 1.5 mM and 0 mM external Ca^2+^ respectively [1,2,20,21,22,23,24,25,26,27,28,29]. Moreover, studies using an array of different techniques such as blocking anti-TRPC antibodies (e.g., T1E3), TRPC1^−/−^ mice, molecular knockdown (e.g., siRNA), and pharmacology (e.g., the selective TRPC1/C4/C5 inhibitor Pico145) [30,31] indicate that these SOCs are composed of a heteromeric TRPC1/C5 molecular template, which can also involve other TRPC subunits depending on the vascular bed [7,20,21,22,23,24,25,26,27,28,29,32,33]. As such, these studies show that TRPC1/C5-based SOCs are functionally expressed in pial arterioles and mesentery artery VSMCs, whereas TRPC1/C5/C6- and TRPC1/C5/C7-based SOCs are reported in coronary artery and portal vein VSMCs respectively. It is predicted that differences in the molecular compositions of these TRPC-based SOCs are reflected in the different properties of TRPC-based SOCs such as rectification, Ca^2+^ permeability and unitary conductance. For example, inclusion of TRPC6 within the TRPC1/C5 template produces biophysical properties which indicate a decreased Ca^2+^ permeability [22], whereas the presence of TRPC7 produces a facilitatory action of IP_3_ on TRPC1-based SOCs [22,34]. Studies using TRPC1^−/−^ VSMCs indicate that TRPC1 is essential for conferring activation by store depletion, and therefore these TRPC1/C5 channel templates are termed TRPC1-based SOCs [25]. These findings fit with the idea that TRPC1 subunits do not form a functional homotetrameric channel [13]. Several studies have indicated that Orai1 proteins are unlikely to be involved in composing SOCs in VSMCs with a native contractile phenotype, with low expression of Orai1 proteins present in these cells ([35,36] and see [37,38] for review of Orai1 proteins in vascular smooth muscle) and biophysical properties of TRPC1-based SOCs and their STIM1-mediated activation mechanisms unaffected in freshly isolated and primary cultured VSMCs from Orai1^−/−^ mice [29].

In contrast to native contractile VSMCs, long-term cultured VSMCs maintained in high serum, conditions that induce a non-contractile synthetic phenotype with proliferative, migrative and growth characteristics, express whole-cell SOCs and store-operated Ca^2+^ entry with similar properties to Orai1-based CRACs such as pronounced inward rectification, inhibition by a Orai1 selective inhibitor, and inhibition by knockdown of Orai1 and STIM1 protein levels but not by knockdown of TRPC1, TRPC4 or TRPC6 proteins [36,37,38,39,40,41,42]. In addition, store-operated whole-cell currents and Ca^2+^ entry are unaffected in cultured VSMCs maintained in high serum conditions from TRPC1^−/−^ mice [43] further suggesting the involvement of Orai1-based CRACs in these VSMCs. In contrast, TRPC1 has also been proposed to be involved in regulating SOCs present in synthetic VSMCs, perhaps by regulating Orai1-based CRACs, and it is suggested that increase in the expression and function of TRPC1 may be an important trigger in the development of the cellular switch from a contractile to synthetic phenotype [37,38].

These complexities mean that when studying SOCs in VSMCs it is essential to clearly define cell phenotype of VSMCs. In this regard, when investigating SOCs in native contractile VSMCs it is advantageous to use freshly isolated cells whenever possible, and when required only use primary culture cells maintained in low serum conditions for as short a time as possible.

## 3. Activation Mechanisms of TRPC1-Based SOCs in Native Contractile VSMCs

The following sections discuss our recent findings that TRPC1-based SOCs in freshly isolated and primary cultured VSMCs with a native contractile phenotype exhibit a complex activation mechanism, in which interactions between PKC-dependent phosphorylation of TRPC1 and phosphatidylinositol 4,5-bisphosphate (PIP_2_) regulated by myristoylated alanine-rich C kinase (MARCKS) are obligatory gating partners, with these mechanisms coupled to store depletion by a novel STIM1-mediated Gq-PLCβ1 pathway.

### 3.1. PKC Activity and PIP_2_ have Obligatory Roles in Activation of TRPC1-based SOCs

There are several lines of evidence which indicate that PKC activity and PIP_2_ are important in activating TRPC1-based SOCs. The stimulation of whole-cell and single channel TRPC1-based SOCs is significantly reduced by PKC inhibitors and anti-PIP_2_ antibodies [22,25,44]. In support of these results, PKC activators (diacylglycerol analogues, phorbol esters, PKC catalytic subunits) and exogenous application of diC8-PIP_2_ (a water-soluble form of PIP_2_) stimulate TRPC1-based SOCs in WT but not in TRPC1^−/−^ VSMCs [22,25,44]. Interestingly, PKC-stimulated activation of TRPC1-based SOCs are prevented by anti-PIP_2_ antibodies and pharmacological agents such as wortmannin to deplete PIP_2_ levels, whereas diC8-PIP_2_-activated TRPC1-based SOCs are reduced by PKC inhibitors [44]. Moreover, store depletion induces PKC-dependent phosphorylation of TRPC1 and increases association between PIP_2_ and TRPC1 [26,27,28,44]. Together, these results indicate that interactions between PKC activity and PIP_2_ have obligatory roles in activation of TRPC1-based SOCs; PKC cannot activate TRPC1-based SOCs without PIP_2_, and vice versa. This supports the hypothesis that PIP_2_ is the activating ligand of TRPC1-based SOCs and that store-operated PKC-dependent phosphorylation of TRPC1 is required for this opening mechanism to occur.

These proposed roles of PKC and PIP_2_ on TRPC1-based SOCs are different to their roles in the activation of TRPC3/C6/C7-based ROCs in native contractile VSMCs (see [23,24] for comprehensive reviews of activation mechanisms of TRPC channels). It is well established that this subgroup of TRPC channels are activated by receptor-mediated generation of DAG which leads to channel opening via PKC-independent mechanisms, with PKC causing channel inhibition. In addition, the role of PIP_2_ on TRPC3/C6/C7-based ROCs is unclear with both inhibitory and excitatory actions proposed [45,46]. These findings further indicate that TRPC1-based SOCs and TRPC3/C6/C7-based ROCs form distinct channel structures with differing activation mechanisms, and likely distinct functions in VSMCs.

Important omissions from our understanding are what PKC isoform(s) is involved, which amino acid(s) within TRPC1 protein structure is phosphorylated, and how a PKC-dependent phosphorylation process alters interactions between TRPC1 and PIP_2_. The PKC family comprises of at least 11 serine/threonine kinases divided into three groups according to their basic structure and activation requirements: conventional PKC isoforms (α, βI, βII and γ) require both Ca^2+^ and diacylglycerol (DAG), novel PKC isoforms (δ, ε, η and θ) require DAG but are Ca^2+^-insensitive, and atypical PKC isoforms (ζ, ι and λ) are activated by lipid mediators such as phosphatidylserine and do not require Ca^2+^ or DAG (see [47] for review of the role of PKC isoforms in vascular smooth muscle). Our results indicate that stimulation of TRPC1-based SOCs and PKC-dependent phosphorylation of TRPC1 by store depletion requires PLCβ1 activity, and that DAG analogues activate TRPC1-based SOCs through a PKC-dependent mechanism [9,23,24,27]. Moreover, we have shown that TRPC1-based SOCs are activated by store depleting agents which are likely to increase (e.g., CPA), decrease (e.g., the high affinity cell-impermeable and -permeable Ca^2+^ chelators BAPTA and BAPTA-AM), or produce little change in Ca^2+^_i_ (e.g., the low affinity cell-permeable Ca^2+^ chelator TPEN). These findings suggest that the PKC isoform involved is likely to require DAG but is Ca^2+^-insensitive, which are characteristics of the novel group of PKC isoforms. Our preliminary data indicate that PKCδ is the most highly expressed novel PKC isoform in VSMCs, and that selective PKCδ inhibitory peptides and knockdown of PKCδ using morpholino sequences prevent activation of TRPC1-based SOCs [31].

Interestingly, the predication of PKCδ-dependent phosphorylation sites within the TRPC1 sequence using GPS 3.0 reveals five intracellular serine residues, with Ser619 and Ser752 at the C-terminal domain of potential significance as both these sites are close to a known PIP_2_-binding domain [48]. It is therefore possible that PKCδ-dependent phosphorylation of these sites increases PIP_2_ affinity leading to increased binding to TRPC1 and channel opening. Previous studies have highlighted that protein kinase A (PKA), protein kinase G (PKG), and calmodulin kinase II (CaMKII) have inhibitory actions on TRPC1-based SOCs in VSMCs [49,50,51], and it may be that phosphorylation of serine/threonine amino acids by these kinases reduce TRPC1 and PIP_2_ interactions by lowering PIP_2_ affinity. Similar roles for kinase activities in modulating lipid-protein interactions are well-established in the regulation of different K^+^ channel subtypes [52].

### 3.2. Interactions Between PKC Activity and PIP_2_ are Regulated by MARCKS

An important question is how PKC-dependent phosphorylation regulates PIP_2_ gating of TRPC1-based SOCs when physiological activators of SOCs involve stimulation of Gq-coupled receptors which drive PLC activity and PIP_2_ hydrolysis. To provide answers to this question, we investigated the role of MARCKS in activation of TRPC1-based SOCs. MARCKS is a membrane-bound PIP_2_-binding protein that acts a PIP_2_ buffer, which can provide a distinct pool of PIP_2_ at the plasma membrane that is protected from breakdown by PLC thus enabling this phospholipid to be released in a coordinated manner into the local environment [53,54]. Using a combination of electrophysiological, co-immunoprecipitation, and PIP_2_-binding dot-blot assays, we showed that in unstimulated conditions MARCKS is bound to TRPC1, with PIP_2_ predominately associated with MARCKS and not TRPC1 [26]. Upon stimulation by store depleting agents MARCKS dissociates from TRPC1, which leads to PIP_2_ being released and associated with TRPC1 to cause channel opening. Importantly, both dissociation of MARCKS from TRPC1 and redistribution of PIP_2_ from MARCKS to TRPC1 are stimulated by phorbol esters and prevented by PKC inhibitors indicating that PKC activity is central to these mechanisms. It will be important to ascertain how receptor stimulation and store-depleting agents couple to these actions of MARCKS. A potential idea is that stimulation of CaM is involved, as this Ca^2+^-binding protein is known to bind to MARCKS and to cause dissociation from the plasma membrane and PIP_2_ release [53,55]. Furthermore, CaM is known to activate TRPC1-based SOCs in native contractile VSMCs [50].

### 3.3. Store Depletion Activates a Gq-PLCβ1 Pathway Involved in Activation of TRPC1-Based SOCs

For a channel to be defined as store-operated, it is essential to understand how store depletion is coupled to channel opening. Therefore, a central question surrounding the activation of TRPC1-based SOCs is how store depletion couples to PKC-dependent phosphorylation of TRPC1 and opening by PIP_2_? A crucial finding was the discovery that store depletion activates a Gq-PLCβ1 pathway which drives the PKC-dependent phosphorylation of TRPC1 [27]. Our studies showed that the G-protein inhibitor GDP-β-S, anti-Gq antibodies (but not anti-Gi), the PLC inhibitor U73122, and PLCβ1 shRNA inhibited activation of TRPC1-based SOCs. In addition, store-operated PKC-dependent phosphorylation of TRPC1 was inhibited by U73122 and PLCβ1 shRNA. A significant finding was that store depleting agents induced PLC activity measured using GFP-PLCδ1-PH, a fluorescent biosensor for PIP_2_ and IP_3_ ([56,57,58] and reviewed in [59]) in primary cultured VSMCs which was prevented by a PLC inhibitor and PLCβ1 shRNA. In support of these findings, co-immunoprecipitation and proximity ligation assays (PLA) demonstrated that store depletion induced interactions between TRPC1, Gq and PLCβ1 at the plasma membrane.

### 3.4. STIM1 Couples Store Depletion to Gq-PLCβ1 Activity to Stimulate TRPC1 SOCs

Next, we investigated how store depletion is coupled to the formation and activation of this Gq/PLCβ1/PKC/TRPC1 signal transduction pathway. An obvious candidate was STIM1, since along with its classically described role in activating Orai1-based CRACs it has been implicated in activating TRPC-based SOCs (see [13] for review). Moreover, it is known that that STIM1 has diverse cellular partners including ion channels [12,13,15,16,17,18,60,61], SR and plasma membrane ATPases [62,63], and adenylate cyclase [64] and therefore it seemed a reasonable idea that STIM1 may couple to Gq/PLCβ1 activity.

We demonstrated that whole-cell and single channel TRPC1-based SOCs and store-operated PLCβ1 activity were inhibited by shRNA STIM1 and were absent in TRPC1^−/−^ VSMCs [28]. In addition, store-operated PKC-dependent phosphorylation of TRPC1 was greatly reduced by shRNA STIM1. Moreover, STIM1 was required for store-operated interactions between Gq, PLCβ1 and TRPC1, and TRPC1 was essential for store-operated interactions between Gq, PLCβ1, and STIM1. These findings provide strong evidence that STIM1 is an essential molecule in activation of TRPC1-based SOCs, and importantly it indicates that store-operated STIM1-TRPC1 interactions (measured using PLA assays which imply that these interactions occur within 40 nm) form the structural basis for stimulation of the Gq/PLCβ1 pathway required for PKC-dependent TRPC1 phosphorylation and channel opening by PIP_2_.

As expected, store depletion induced translocation of STIM1 from the cytosol to the plasma membrane where it formed discrete puncta and interactions with TRPC1 using immunocytochemical and PLA techniques, which were not dependent on downstream molecules in the signal pathway such as PLCβ1 [28]. Interestingly, in the absence of TRPC1, store depletion still induced translocation of STIM1 from cytosol to the plasma membrane, but STIM1 formed an even distribution throughout the plasma membrane and not discrete puncta. This suggests that TRPC1 may be essential for coordinating the response of STIM1 to store depletion.

An interpretation from these studies is that STIM1-TRPC1 interactions, possibly involving other unknown molecules, act as a cellular activator of Gq subunits, and that this leads to interactions with, and activation of, PLCβ1. In essence STIM1-TRPC1 interactions behave like Gq-coupled receptors or guanine exchange factors (GEFs). In the future, it will be important to investigate structural interactions between STIM1 and TRPC1 and discover where Gq subunits binds. Initial work might focus on the CRAC-activating (CAD) and polybasic domains which have been linked to binding and activation of TRPC1 by protein-protein interactions and electrostatic interactions respectively [13,15,16,17,18].

Interestingly, differential blocking effects of N-terminal and C-terminal anti-STIM1 antibodies on TRPC1-based SOCs using whole-cell and inside-out patch clamp recordings may suggest that store-operated STIM1/TRPC1 interactions lead to STIM1 acting as a transmembrane protein at the plasma membrane [28]. In this configuration, the N-terminal domain of STIM1 is now present at the extracellular surface of the plasma membrane. Perhaps this orientation of STIM1-TRPC1 interactions has implications for association with Gq subunits in native contractile VSMCs. A similar role for STIM1 has been previously described in mediating store-operated conductances [65]. However, it seems unlikely that the source of STIM1 involved in activating TRPC1-based SOCs resides at the plasma membrane, as with activation of arachidonic acid-regulated channels (ARC) composed of Orai1/Orai3 subunits [66], since our data indicates that store depletion induces translocation of STIM1 from the cytosol to the plasma membrane.

### 3.5. Activation Mechanisms of TRPC1-Based SOCs are Independent of Orai1

There is substantial evidence that TRPC1, Orai1 and STIM1 have important roles in mediating store-operated Ca^2+^ entry pathways in VSMCs with a synthetic phenotype, although it is unclear whether Orai1 and TRPC1 interact together or form separate STIM1-mediated ionic mechanisms (see earlier). In addition, it is unclear whether Orai1 is required for activation of TRPC1-based SOCs in native contractile VSMCs. In our recent study, we showed that the properties of store-operated whole-cell and single channel currents in freshly isolated and primary cultured native contractile WT and Orai1^−/−^ VSMCs were similar to previously described TRPC1-based SOCs, and that store-operated STIM1-mediated PLCβ1 activity and STIM1-TRPC1 interactions were unaffected in Orai1^−/−^ cells [29]. These findings provide significant evidence that Orai1 proteins are not required for the molecular composition or activation mechanisms of TRPC1-based SOCs in contractile VSMCs. These results support the findings that Orai1 expression is very low in native contractile VSMCs [35,36,37,38]. A caveat is that Orai proteins are composed of three subtypes, Orai1-3 [12], and therefore Orai2 and Orai3 may be involved in TRPC1-based SOCs in native contractile VSMCs. However, whole-cell store-operated conductances with the distinct characteristics of Orai proteins, such as such inward rectification, and reversal potential greater than +50 mV have not been identified in native contractile VSMCs from WT, Orai1^−/−^ or TRPC1^−/−^ preparations [25,29].

It should be noted that other studies using pharmacological and molecular techniques have implicated Orai1 and Ca^2+^-independent phospholipase A_2_ (iPLA_2_) in store-operated conductances in native and primary cultured contractile VSMCs [67]. These studies propose that store depletion linked to STIM1-mediated release of a calcium influx factor (CIF) from the SR activates Orai1 through an iPLA_2_-mediated mechanism involving production of lysophospholipids (see [68] for review). In addition, Orai1 has been proposed alongside TRPC1 to regulate store-operated vascular contractility through interactions with voltage-gated Ca^2+^ channels ([69] and reviewed in [70]). The differences between these findings to those described above are unclear and may reflect differences between SOCs in different vascular beds and perhaps different cell isolation or culturing conditions. However, before these ideas can be truly accepted it will be essential to resolve the identity of CIF and understand why proposed Orai1-sensitive currents in contractile VSMCs have such a linear rectification [67]. For example, do contractile VSMCs express an Orai1 splice variant with very different properties to established Orai-based CRACs? In addition, a difficult finding to explain is why store-operated conductances are absent in native contractile VSMCs from TRPC1^−/−^ mice [25]. Moreover, involvement of iPLA_2_ has been inferred in many studies using the proposed inhibitor bromoenol lactone (BEL), which has also been shown to block heteromeric TRPC1/C5, TRPC5, and TRPC6 channels and VGCCs [71].

## 4. Physiological Significance of TRPC1-Based SOCs Activation Pathway

To avoid complications from receptor-operated pathways, most studies use agents that selectively deplete SR Ca^2+^ stores (e.g., CPA, BAPTA) instead of receptor stimulation to study SOCs (see Introduction). However, it is important to examine whether proposed store-operated mechanisms are involved in activation of SOCs by physiological receptor stimulation. This is particularly important for TRPC1-based SOCs in native contractile VSMCs as these channels also behave as ROCs, and so may be activated by distinct store-independent and -dependent pathways [1,2]. Previous studies have shown that noradrenaline, Ang II and ET-1-activated ET_B_ receptor stimulation induce TRPC1-based SOCs via a PLC-mediated pathway that requires PKC activity and PIP_2_ for channel opening in portal vein, mesenteric artery and coronary artery VSMCs respectively [8,9,10,11,23,72]. To investigate whether these receptor-mediated activation pathways of TRPC1-based SOCs might involve our proposed store-operated STIM1/Gq/PLCβ1 pathway, we showed that noradrenaline-activated TRPC1-based SOCs were greatly reduced by knockdown of PLCβ1 and STIM1 and that noradrenaline also induced interactions between STIM1 and TRPC1, Gq, and PLCβ1 [27,28]. Moreover, noradrenaline induced PKC-dependent phosphorylation of TRPC1 and activation of TRPC1-based SOCs by regulating MARCKS-TRPC1-PIP_2_ interactions similar to those produced by store depleting agents [26]. These findings highlight that physiological receptor stimulation is likely to activate TRPC1-based SOCs through the store-operated STIM1/Gq/PLCβ1/PKC pathway and also via a store-independent pathway involving a Gq/PLC/PKC pathway. It may be that receptor stimulation switches between these two modes to activate TRPC1-based SOCs according to concentration of the physiological agonist. For example, higher vasoconstrictor concentrations may be more likely to produce substantial IP_3_-mediated depletion of SR Ca^2+^ stores and activation of TRPC1-based SOCs by store depletion.

It is likely that certain receptors only activate TRPC1-based SOCs via store-independent pathways. In native contractile coronary artery VSMCs, ET-1-activated ET_A_ receptor stimulation induces TRPC1-based SOCs through a Gβγ-protein-phosphoinositol 3-kinase (PI3K)-mediated pathway, with PIP_3_ formation thought to both induce PKC activity and act as the channel activating ligand; a pathway which is unlikely to involve IP_3_-mediated depletion of SR Ca^2+^ stores [11,72]. Therefore, TRPC1-based SOCs in coronary artery VSMCs are activated by ET_A_ and ET_B_ receptor stimulation through separate store-independent and -dependent pathways respectively, both requiring obligatory roles of PKC activity and phospholipids [11,66].

Interestingly, the knockdown of PLCβ1 and STIM1 had little effect on noradrenaline-stimulated PLC activity measured using the GFP-PLCδ-PH biosensor, although it was inhibited by U73122 a general PLC isoform inhibitor [27,28]. These findings support significant physiological relevance to our results, as it suggests that stimulation of Gq-protein-coupled receptors and IP_3_-mediated depletion of SR Ca^2+^ stores are likely to activate two different Gq-PLC pathways mediated by distinct PLC isoforms. To test this hypothesis, it would be important to identify the PLC isoform linked to α_1_-adrenoceptor stimulation. The critical role of MARCKS in stimulation of TRPC1-based SOCs is further highlighted when considering the physiological significance of our proposed activation pathway. Stimulation of Gq-protein-coupled receptors and IP_3_-mediated depletion of SR Ca^2+^ stores will both induce Gq-PLC activities leading to PIP_2_ hydrolysis. Therefore, MARCKS provides the essential role of a buffering a separate pool of PIP_2,_ which is protected from PLC-mediated hydrolysis enabling the phospholipid to be available for channel opening.

## 5. Summary

**Figure 1:**
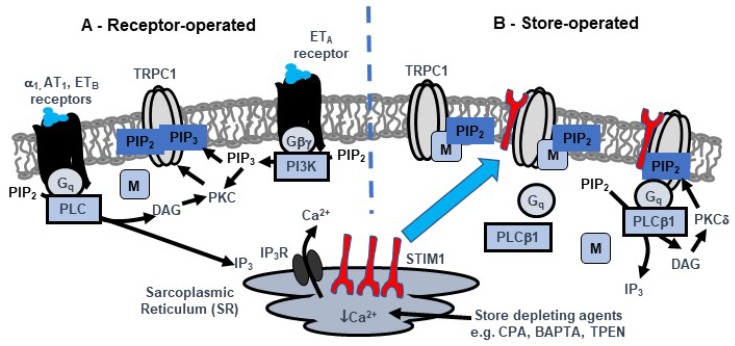
Proposed model of TRPC1-based store-operated channels (SOCs) in native contractile vascular smooth muscle cells (VSMCs)**.** A, Receptor stimulation of distinct Gq-PLC and Gβγ-PI3K-PIP_3_ pathways lead to PKC-dependent phosphorylation of TRPC1 and channel opening by PIP_2_ and PIP_3_ respectively. Local levels of PIP_2_ involved in channel activation is controlled by MARCKS (M). B, Store depletion by receptor-mediated IP_3_ generation and store depleting agents such as CPA, BAPTA and TPEN activate TRPC1-based SOCs through a STIM1-TRPC1-mediated pathway. In unstimulated cells, SR Ca^2+^ stores are full and TRPC1-based SOCs are in a closed state. In this configuration, channels are associated with MARCKS which buffers local PIP_2_ levels and do not interact with Gq, PLCβ1 or STIM1. Following SR Ca^2+^ depletion, STIM1 (red) is activated and translocates from the SR to the plasma membrane where it interacts with TRPC1. Formation of STIM1-TRPC1 interactions enable binding and activation of Gq and PLCβ1 activity, PIP_2_ hydrolysis, DAG generation, stimulation PKCδ and PKC-dependent phosphorylation of TRPC1. This leads to dissociation of MARCKS from TRPC1 and release of PIP_2_ (previously protected from PIP_2_ hydrolysis) into the local environment where it acts as the activating ligand.

This review highlights recent findings that SOCs in native contractile VSMCs are mediated by TRPC1-based SOCs, which have very different biophysical properties to the well-characterized channels formed by Orai proteins, including archetypal Orai1-based CRACs. Moreover, Figure 1 describes a novel activation pathway for these TRPC1-based SOCs proposed from our recent studies, in which SR Ca^2+^ store depletion by receptor-induced IP_3_-mediated generation or store depleting agents such as CPA and BAPTA stimulate STIM1 to translocate to the plasma membrane where it interacts with TRPC1 subunits to form a receptor for Gq G-protein subunits which induces PLCβ1 activity, production of DAG, and PKC activity. PKC-dependent phosphorylation of TRPC1 increases PIP_2_ binding and channel opening. The interactions between TRPC1 and PIP_2_ are regulated by MARCKS, a plasma membrane PIP_2_-binding protein bound to TRPC1 at rest, which upon store depletion dissociates from TRPC1 and releases PIP_2_ into the local environment where the phospholipid acts as the gating ligand. Figure 1 also depicts our current understanding of how receptor stimulation by vasoconstrictors activates TRPC1-based SOCs in contractile VSMCs by store-independent pathways involving PKC activity and phospholipids.
